# Exploring public health researchers’ approaches, barriers, and needs regarding dissemination: A mixed-methods exploration

**DOI:** 10.1017/cts.2024.527

**Published:** 2024-04-22

**Authors:** Alicia Nieuwland, Jillian Rose O’Mara, Joreintje Dingena Mackenbach

**Affiliations:** 1 Epidemiology and Data Science, Amsterdam UMC location Vrije Universiteit Amsterdam, Amsterdam, The Netherlands; 2 Amsterdam Public Health research institute, Amsterdam, The Netherlands; 3 Public and Occupational Health, Amsterdam UMC location Universiteit van Amsterdam, Amsterdam, The Netherlands

**Keywords:** Dissemination and implementation science, knowledge transfer, research communication, translation gap, valorization

## Abstract

**Background:**

Although there is growing attention to research translation, dissemination practices remain underdeveloped. This study aimed to gain insights into the dissemination approaches, barriers for dissemination, and needs for dissemination support of public health researchers of the Amsterdam Public Health (APH) research institute.

**Methods:**

A concurrent mixed-methods design was used, collecting quantitative and qualitative data through a survey and qualitative data from interviews. Researchers of the Health Behaviors and Chronic Diseases (HBCD) research line of APH were approached via email with a link to an online survey. For the interviews, we aimed to balance researchers in terms of career phase and position. Data were analyzed through descriptive statistics and thematic content analysis.

**Results:**

HBCD researchers primarily rely on traditional approaches for dissemination, e.g. academic journals (93%), conferences (93%), and reports to funders (71%). Social media (67%) was also frequently mentioned. Dissemination is often prioritized late due to time constraints and competing priorities. Researchers mentioned a lack of time, money, knowledge, and skills but also limited awareness of available support as barriers. A need for more resources, education, and a shift in mindset was expressed, suggesting a comprehensive inspiring platform and stronger in-house connections as solutions.

**Conclusion:**

HBCD researchers emphasized the importance of dedicated time and budget for dissemination, as well as other forms of institutional support. Overall, there is a need for a shift in mindset, more educational initiatives, greater integration of dissemination into researchers’ roles, the establishment of a comprehensive inspiring platform, and stronger in-house connections to support dissemination efforts.

## Introduction

Effective translation of research findings into practice has the potential to optimize health prevention, health promotion, and health care practices [1]. Nonetheless, too frequently, attempts to translate effective preventative measures into widespread use have been disorganized, fragmented, and underfunded [1]. Consequently, the field of dissemination and implementation science has emerged to address this translation gap and to increase the uptake and the impact of research findings [2]. Within this field, dissemination research focuses on the understanding of factors that lead to the broad use of research findings [3] (“helping it happen” [4]), while implementation research focuses more on the methods, processes, and frameworks to promote uptake into routine practices in specific settings [3] (“making it happen” [4]). In light of the current study, we are primarily interested in dissemination, which we defined as “the broad range of activities used to spread scientific knowledge to a target audience through planned strategies” [5].

There has been great progress made within the dissemination field [6], with a wide majority of researchers valuing dissemination and many funding organizations mandating a detailed plan for the dissemination of research findings [7]. However, specific guidance on how to effectively carry out dissemination is lacking [7]. Ineffective dissemination of public health research findings to the target audience can lead to missed opportunities for health promotion, disease prevention, and a sustained burden of disease [1], although it should be noted in some cases dissemination activities may not be appropriate. In addition, ineffective dissemination wastes project funding and researchers’ efforts [8,9]. The dissemination efforts of public health researchers are often still suboptimal, potentially due to a lack of resources and lack of clarity about the party responsible for the dissemination of research findings [8,10]. Indeed, one-third of public health researchers in the United States (US) rate their dissemination efforts as poor [8]. In a similar study in the United Kingdom (UK), 10% of researchers of publicly funded applied and public health research rated their efforts as poor [11]. This study emphasized that UK researchers are in need of better guidance on how to plan, resource, and facilitate their dissemination activities [11]. As such, addressing deficiencies in dissemination and increasing the uptake of research-based knowledge into practice is essential.

To improve dissemination practices in public health, it is important to understand current approaches, barriers to dissemination, and needs for dissemination support. However, the existing literature lacks fundamental studies on dissemination efforts, such as a knowledge of researchers’ attitudes, practices, and the factors that influence the dissemination of research findings [12]. One UK study showed that public health researchers predominantly rely on academic journals (99%) and academic conferences (81%) as their primary dissemination methods [11]. These dissemination methods have been proven inadequate in meeting the unique and ever-changing needs of adopters [7]. Other literature has highlighted barriers to dissemination, including a lack of training, funding, institutional support, and time [12]. However, to our knowledge, no study has systematically explored the current approaches, barriers to dissemination, and the needs for dissemination support in a public health researcher population in the Netherlands.

Given the importance of dissemination in the public health field, our purpose was to (a) gain insights into the dissemination approaches of public health researchers, (b) identify barriers public health researchers encounter when disseminating their research findings, and (c) explore public health researchers’ needs for dissemination support. This was investigated within the Amsterdam Public Health (APH) research institute, specifically focusing on researchers in the Health Behaviors & Chronic Diseases (HBCD) research program.

## Materials & methods

### Design

A concurrent mixed-methods approach was used, collecting quantitative and qualitative data through an online survey with closed and open-ended questions and qualitative data from interviews. The quantitative survey was able to reach a larger group of participants through standardized questions relevant to the study, while the interviews gave opportunities for a smaller group of participants’ perceptions toward dissemination via semi-structured interviews [13]. The research design was submitted to the Amsterdam University Medical Centre (UMC) ethical committee [METC number 2023.0230], which determined it was not subject to the Medical Research Involving Human Subjects Act (Wet Medisch-Wetenschappelijk onderzoek met mensen (WMO)) approval.

### Research model

The Knowledge-to-Action (KTA) framework of Graham and colleagues [14] guided the conceptualization and analysis of this study. This framework consists of a knowledge creation cycle and an action cycle. The action cycle consists of several phases, including the problem identification, the adoption of knowledge to local context, the assessment of barriers to knowledge use, the selection, tailoring and implementation of interventions, the monitoring of knowledge use, the evaluation of outcomes, and the sustainment of knowledge [14]. In reality, these phases may be complex and fluid, and can either follow or happen simultaneously with the knowledge-creation cycle [14]. The KTA framework is frequently used as a founding theory in dissemination studies [15] and provides a broad overview of the dissemination process for researchers.

### Study population

The study population consisted of HBCD researchers within APH. These researchers can be affiliated with the following institutions: Vrije Universiteit Amsterdam (VU), University of Amsterdam (UvA), and the Amsterdam UMC (location VUmc or AMC). The link to the online survey was sent out by a general APH email account to all 242 HBCD researchers. Researchers could participate if they provided informed consent (opt-in) and were proficient in the English language. HBCD researchers involved in the design of this study were excluded. For the interviews, non-probability purposive sampling was used whereby participants were invited based on their career phase, position, and institution. These participants were approached via email or in person. Researchers could participate in the interviews if they provided informed consent (opt-in) and were proficient in English or Dutch.

### Procedure

The online survey was accessible via Lime Survey for a period of 3 weeks (from May 9, 2023 to May 31, 2023), with a reminder sent after 1.5 weeks. The survey outcomes were anonymous and covered a wide range of topics including but not limited to the motivations for dissemination, commonly used methods/strategies, dissemination planning, and experienced barriers. Table 1 includes an overview of the topics, the survey questions, and their operationalization. The survey was based on a survey by Brownson and colleagues [10] which aimed to describe the dissemination practices of public health researchers in the US. Changes were made in the survey to fit the design of this study, including shortening the survey, removing project-specific questions, and adding open questions and new media answering categories. Additionally, a pilot survey was conducted among a representative sample of HBCD-members leading to final changes in the survey. The survey incorporated different types of questions, including binary (yes/no/not sure), categorical (very important/important/somewhat important/not important/not sure) (always/usually/sometimes/rarely/never/not sure), and open-ended questions (see supplementary material 1). Open answers were re-coded as existing answering categories if possible and otherwise qualitatively analyzed. The categorical responses were coded based on the scale categories, sometimes merging two (similar) categories.

**Table 1.** Survey operationalization based on the survey of Brownson and colleagues [10]

Question	Variable type	Operationalization
Please pick your institution*	Categorical; nominal	Amsterdam UMC^b^ VUmc^c^
*Note: HBCD^a^-researchers can come from these four different institutes.*		
		Amsterdam UMC AMC^d^
		Vrije Universiteit Amsterdam (VU)
		University of Amsterdam (UvA)
Please enter your role/position at this institution*	Open	
**Background perceptions on dissemination**		
Is the dissemination of research findings part of your role?*	Binary	Yes
		No
		Maybe
Do you think the dissemination of research findings should be part of your role?*	Binary	Yes
		No
		Not sure
How important to your own research is dissemination?*	Categorical; ordinal	Very important
		Important
		Somewhat important
		Not important
		Not sure
How important is dissemination to the work of your research group?*	Categorical; ordinal	Very important
		Important
		Somewhat important
		Not important
		Not sure
**Guidance for dissemination**		
Is there a dedicated person or team responsible for dissemination-related activities within your organization and do you make use of the support they offer?*	Binary	Yes, there is. And yes, I make use of their support.
		Yes, there is, I don’t make use of their support.
		No, there is not.
		Not sure
Does your research group have a formal communication/dissemination strategy?* Think for instance about certain policies and/or guidelines within your research group concerning dissemination.	Binary	Yes
		No
		Not sure
Do you ever refer to guidance documents or use a framework to plan dissemination-related activities?* For example, think about the guidance documents on the APH webpage about dissemination.	Categorical; ordinal	Always
		Usually
		Sometimes
		Rarely
		Never
		Not sure
**Motivation for dissemination**		
Why do you disseminate the findings of your research?*	Categorical; nominal *Note: multiple answers possible*	To raise awareness of the findings
		To stimulate discussion/debate
		To influence policy
		To influence practice
		To transfer research to practice
		To justify public funding
		To attract future
		funding
		To raise the
		organizational profile
		To improve your own communication
		To promote public understanding of science
		To satisfy contractual obligations
		other (please give details below)
Please give details on the question above if needed.	Open	
Which of the reasons given above for disseminating the findings of your research are the most important?* Please state your top three.	Open categorical; ordinal	most important:
		second most important:
		third most important:
**Planning of dissemination activities**		
At what stage in the research process do you usually plan dissemination-related activities?*	Categorical; nominal *Note: multiple answers possible*	When the research is being formulated
		At the proposal stage
		During the research process
		At the draft report stage
		At the final report stage
		At all stages of the process
		Question not applicable
As part of your research dissemination, do you ever think about who needs to know about the findings and/or who is most likely to be influenced by them or will influence others?*	Categorical; ordinal	Always
		Usually
		Sometimes
		Rarely
		Never
		Not sure
As part of your research dissemination, do you ever consider how audiences or group would like to reach, access, read, and use research findings?*	Categorical; ordinal	Always
		Usually
		Sometimes
		Rarely
		Never
		Not sure
**Dissemination activities and approaches**		
What methods do you usually use to disseminate research findings? Please pick all that apply*	Categorical; nominal *Note: multiple answers possible*	Academic journals
		Professional journals
		Report to funders
		Full report
		Summary report
		Press releases
		Newsletters
		Policy briefing paper
		Email alerts
		RSS feeds
		Targeted mailings
		Conferences
		Seminars and/or workshops
		Face-to-face meetings
		Networking
		Media interviews
		Social media posts
		Research registers
		Vlogs
		Podcasts
		Poster
		Infographics
		Factsheets
		Other (please give details below)
Please give details on the question above if needed.	Open	
Of the methods you use to publish and disseminate the research findings, which do you think generally have the most impact and why?*	Open	
**Barriers for dissemination**		
Are there any methods of disseminating research findings that you would like to use but are unable to do so?*	Binary	Yes
		No
		Not sure
If your answered yes above, please provide details including what you would need in order to use those methods	Open	
Is there anything else you can think of what would enhance the impact of your research?* If not please type no.	Open	
**Dissemination efforts**		
Do you every evaluate the impact of the research?*	Categorical; ordinal	Always
		Usually
		Sometimes
		Rarely
		Never
		Not sure
Overall, how do you rate your current dissemination activities?*	Categorical; ordinal	Excellent
		Good
		Adequate
		Poor
		Not sure

* Mandatory question.

a Health Behaviors & Chronic Disease (research program of the Amsterdam Public Health research institute).

b University Medical Center.

c Vrije Universiteit medical center.

d Amsterdam Medical Center.

The interviews were conducted by one researcher using a semi-structured interview guide. This guide was based on the KTA framework and included topics such as adopting knowledge to local context, barriers to knowledge use, selecting, tailoring, and implementing dissemination strategies (see supplementary material 2). A pilot interview was conducted with a representative researcher to test the structure and flow of the topic guide, resulting in minor refinements. Prior to participation, interview participants received an informed consent form (opt-in). The interviews lasted approximately 30-45 minutes, with an average duration of 41:09 minutes. Interviews were conducted either face-to-face or online using Microsoft Teams, depending on the participant’s preference. Interviews were conducted in either English or Dutch and transcribed using a non-verbatim approach. To ensure data quality, a member check was conducted, summarizing the main topics discussed in the interviews and confirming with participants whether the researcher’s understanding aligned with their intended message. To ensure data security, recordings and all other data were stored on a secured server of Amsterdam UMC and participant characteristics were excluded from final reporting.

### Data analysis

The survey generated quantitative and qualitative data. This report only includes data relevant to the research aims. There were some missing values, but descriptive statistics were provided for each question separately, using the maximum available data. Descriptive analyses generating means, standard deviations, and percentages were done in SPSS. Qualitative data from the survey were thematically coded in MAXQDA based on the codes established from the interview data.

The interview transcripts were analyzed using MAXQDA. The analysis involved reading the transcripts and employing both inductive and deductive coding. Deductive coding was used to identify themes based on the KTA framework, while inductive coding revealed themes beyond the scope of the framework. The coding process encompassed three stages: open, axial, and selective coding [16]. Open coding adhered closely to participants’ statements, whereas axial coding involved grouping these codes into broader themes and subcategories, followed by selective coding to examine the coherence among the established themes [16]. The coded segments were checked by a second researcher.

Finally, the data from the survey and the interviews were combined through narrative integration by taking into account any instances where the results from the different methods appeared to be in conflict, in agreement, or gave complimentary information on similar topics [17]. Additionally, the follow-the-thread method was used where essential ideas and themes from one data collection method were followed throughout the other data collection method [17].

### Validity, reliability, and reflexivity

Face validity was established through both in person and online video interviews. Construct validity was ensured by using the KTA framework as the basis for adjusting the survey and designing the interview guide, ensuring that they measured the intended constructs and avoided measuring unrelated factors [13]. The combination of qualitative and quantitative methods, known as data triangulation, further enhanced the validity and reliability of the findings [13]. Additionally, reliability and consistency of measurements were maintained by employing standardized tools and a uniform approach across all interviews, with a focus on researcher neutrality and consistency [18]. Participant-researcher dynamics and contextual influences played a role, and the researcher’s background and supervision team’s characteristics were acknowledged as potential sources of bias [19].

## Results

### Sample characteristics

A total of 58 researchers (response rate = 42%) responded to the survey, of which 42 completed the survey (completion rate = 72%). This resulted in a total response rate of *N* = 42 for each question (Table 2). For the interviews, a total of 11 researchers (*N* = 11) from several different positions, career phases, and institutions were interviewed (Table 3).

**Table 2.** Spread of the survey participants divided by role and institution

		*N*	%
**Career phase & role**			
Early	PhD Student	17	40%
	Post doc researcher	4	10%
	Junior researcher	3	7%
	***N* = 24 (57%)**		
Mid	University teacher	2	5%
	Assistant professor	5	12%
	Associate professor	1	2%
	Intervention developer/researcher	1	2%
	Research associate	1	2%
	***N* = 10 (23%)**		
End	Full professor	4	10%
	Senior researcher	4	10%
	***N* = 8 (20%)**		
**Institution**			
Amsterdam UMC^a^ (VUmc^b^)		24	57%
Amsterdam UMC (AMC^c^)		12	29%
Vrije Universiteit Amsterdam (VU)		6	14%
University of Amsterdam (UvA)		0	0%
**Total *N* = 42**			

a University Medical Center.

b Vrije Universiteit medical center.

c Amsterdam Medical Center.

**Table 3.** Spread of interview participants including, career phases, roles and institution

	Institution	Institutional role
**Career level**		
Early career	University of Amsterdam (UvA)	Post doc researcher
	Vrije University Amsterdam (VU)	PhD candidate
	Amsterdam UMC^a^ – AMC^b^	PhD candidate
Mid-career	Vrije University Amsterdam (VU)	Assistant professor
	Amsterdam UMC - AMC	Assistant professor
	University of Amsterdam (UvA)	Senior researcher / Research associate
	Amsterdam UMC – VUmc^c^	Assistant professor
	Amsterdam UMC - VUmc	Senior researcher / Research associate
End career	Amsterdam UMC - VUmc	Full professor
	Vrije University Amsterdam (VU)	Full professor
	University of Amsterdam (UvA)	Full professor
**Total *N* = 11**		

a University Medical Center.

b Amsterdam Medical Center.

c Vrije Universiteit medical center.

### Background perceptions on dissemination

Participants were familiar with the concept of dissemination. Most made a clear distinction between dissemination to the scientific community and the public and between the dissemination of research findings and the broader dissemination of general knowledge. Dissemination was perceived as very important or important for both researchers’ own research (93%, *N* = 39) as for their research groups (93%, *N* = 39). The significance of dissemination was further emphasized by 95% (*N* = 40) of the survey participants believing that dissemination should be part of their role as researcher. However, 45% (*N* = 19) of the HBCD researchers did indicate the absence of a formal communication/dissemination strategy and 40% (*N* = 17) indicated uncertainty about the communication/dissemination strategies in place.

### Research aim 1: Current dissemination approaches of HBCD researchers

#### Motivations

The most prominent motives identified for dissemination were raising awareness (88%, *N* = 37), influencing policy (79%, *N* = 33), influencing practice (79%, *N* = 33), and transferring research into practice (74%, *N* = 31) (Table 4). These motives were also identified in the interviews. Furthermore, interviewees stated that dissemination is progressively being incorporated as a standard criterion in grant applications. This includes planning for dissemination efforts, budgeting for dissemination, and being more elaborate on what your dissemination efforts will entail. This was highlighted by an interviewee who stated: *“So, in various grant applications, there is an increasing demand for knowledge dissemination and the efforts one will undertake. The standard phrase of ‘we will present it at various scientific and practice-oriented conferences’ is no longer sufficient. There is a growing emphasis on thinking about how we can reach the widest possible audience with our research*.” R10 (UvA).

**Table 4.** Motivations for dissemination as indicated in the survey

Reason		Percentage	Amount
Raise awareness of the findings		88%	37
Influence policy		79%	33
Influence practice		79%	33
Transfer research to practice		74%	31
Stimulate discussion/debate		57%	24
Promote public understanding of science		40%	17
Justify public funding		36%	15
Attract future funding		24%	10
Raise the organizational profile		17%	7
Improve your own communication		12%	5
Other	Increase responses	2%	1
	Scientific dissemination to increase scientific knowledge on a topic	5%	2
	To inform practice	2%	1
	Visibility as a researcher, to establish expert status	2%	1
**Total *N* = 42**			

#### Planning

Around one-third of the HBCD researchers reported in the survey that they plan for dissemination during the proposal stage of their research project. However, the majority of HBCD researchers (52%, *N* = 22) stated that this is done at the final project stage. Interviewees attributed this to the fact that the most significant results tend to emerge at the end of projects, leading to a focus on dissemination during this phase. One interviewee mentioned: *“Often, it is the case that you are at the very end of the research project when significant results emerge. And to be completely honest, the scientific system doesn’t work in your favor at that point. By the time you reach the end, many people are already focused on the next project.”* R2 (VUmc). Interviewees stated that ideally, planning for dissemination should be done at the proposal stage, so that researchers are more likely to engage in dissemination as a natural part of their workflow.

#### Commonly used strategies

Commonly used dissemination strategies include mostly traditional dissemination approaches such as academic journals and conferences (93%, *N* = 39), reporting to funders (71%, *N* = 30), and conducting seminars/workshops (67%, *N* = 28) (Table 5). A survey participant elaborated on the reason for commonly using academic journals stating*: “To reach a large target audience, not bound to time, can be easily referred to/archived for later use.”* Additionally, using social media (67%, *N* = 28) was mentioned as a popular strategy in the survey. This conflicted with the interview data as interviewees expressed that they felt a lack of knowledge when it came to social media but would like to use this more as a dissemination strategy. An interviewee framed this as follows: *“Social media for instance. At the moment I don’t use it. [….] I would like some support for it, how to use it wisely […]’* R10 (VUmc). Additionally, the interviews revealed that engaging with and informing the target group was a common strategy used for generating excitement about research findings. To do this, newsletters, factsheets, and infographics were mentioned as commonly used strategies. Knowledge dissemination through speaking on a topic or giving media interviews was also mentioned in the interviews. In terms of strategies used, no differences were observed in regard to the different career phases included.

**Table 5.** Commonly used dissemination approaches as indicated in the survey

Strategy		Percentage (*N* = 42)	Number
Academic journal		93%	39
Conferences		93%	39
Report to funders		71%	30
Seminars/workshops		67%	28
Social media posts		67%	28
Poster		50%	21
Press releases		45%	19
Newsletters		45%	19
Face-to-face meetings		45%	19
Networking		40%	17
Infographics		40%	17
Factsheets		40%	17
Professional journal		31%	13
Summary report		29%	12
Media interviews		21%	9
Research registers		12%	5
Targeted mailings		12%	5
Full report		10%	4
Policy briefing paper		10%	4
Email alerts		10%	4
Podcasts		7%	3
Vlogs		5%	2
Other	Blogpost	2%	1
	Magazines	2%	1
	Websites	2%	1
RSS feed		0%	0

These dissemination strategies were selected based on what feels right, what has worked in the past, and what fits the research and the researcher. One researcher stated: *“Perhaps there is also a bit of intuition involved in determining what works well for what manuscript or researcher. If you believe something could be important for certain individuals, you engage in discussions and explore the possibilities.”* R5 (VUmc). According to the survey, the majority of HBCD researchers rarely (24%, *N* = 10) or never (40%, *N* = 17) refer to guidance documents or utilize a framework when planning dissemination activities. Only a small percentage usually engages in this practice (7%, *N* = 3).

#### Individual dissemination efforts

In terms of how HBCD researchers score their own dissemination efforts, the largest group rated their efforts as adequate (38%, *N* = 16), followed by 29% (*N* = 12) as poor, 24% (*N* = 10) as good, and 9% (*N* = 4) was unsure about their own efforts. None of the HBCD researchers rated their efforts as excellent. This pattern was also observed in the interviews, with participants describing their dissemination efforts as a work in progress, acknowledging the constraints of insufficient time, knowledge, and resources available for effective dissemination. One interviewee stated when asked about their own dissemination effort: *“In the future (it will be) good, but currently it is still a work in progress. A lot of things are planned, but we are just not there yet.”* R11 (AMC).

### Research aim 2: barriers to dissemination

#### Time & money

The interviews revealed that time is perceived as a significant barrier across the various stages of the dissemination process. For example, in identifying and contacting the right target population, creating dissemination materials, and planning dissemination efforts. The time spent on dissemination detracts from other researcher responsibilities. Therefore, dissemination is often seen as something researchers have to do on the side. One interviewee phrased: “*Yes, it’s actually almost like a separate job. If you really want to do it well, it should be a much more dedicated activity, not just something you do on the side of research projects.”* R2 (VUmc). The outsourcing of dissemination could save time. However, this introduces a cost barrier. Money serves as a constraint in various aspects of the dissemination process, including the compensation for participants’ time, the development of materials, and the outsourcing of expertise.

#### Knowledge & skills

There is also a barrier attributed to a perceived lack of knowledge and skills. Interviewees stated they feel ill-equipped to effectively disseminate their findings, primarily because they did not receive specific training in this area. Consequently, they are hesitant to use certain dissemination strategies, concerned that their lack of expertise may lead to a loss of nuance in conveying their findings to a wider audience. This includes strategies such as the use of social media, podcasts and vlogs, and writing press releases.

#### Different priorities surrounding dissemination

Another barrier emphasized in the interviews was the different levels of priority for dissemination within the various institutions and stakeholders engaged. Dissemination is not regarded as a priority within APH, which can be attributed to the perceived lack of support for and acknowledgment of dissemination efforts, and unclear expectations surrounding the scope of dissemination. As one interviewee stated: *“But I think there can be significant differences between institutions. (..) I believe it would be great if APH could take on a more prominent role, saying: “despite variations between institutions, we expect certain things to be done.””* R9 (VU).

### Research aim 3: needs for dissemination support

#### Solving existing barriers

Regarding time and money, researchers indicated they need more time to spend on dissemination activities and sufficient allocation of budgetary resources to support their dissemination efforts. To solve the knowledge and skills barrier HBCD-researchers expressed a need for more educational activities to enhance their dissemination skills. An interviewee stated: *“Well, I think there should be more guidance overall. (…) We are not trained in this type of communication. So, there are fantastic courses available on writing press releases or managing social media and disseminating information to patients. All of that could be incorporated into training.”* R8 (VUmc). Lastly, to solve the existing barrier surrounding the priority for dissemination, interviewees voiced a need for more institutional support. This could also be seen as a cultural shift that is needed, which can be facilitated by department heads leading by example, paying more attention to dissemination in department meetings, and setting clear expectations surrounding the scope of dissemination.

#### Inspirational platform

Participants suggested that an inspiring platform could be used to address some of the existing needs surrounding dissemination. This platform could offer a menu of different dissemination strategies for different target audiences, based on researchers’ interests. One participant voiced some of the questions such a platform could help address: *“Exactly, something like: “What are the options? Which group does it serve? How do I learn about it? How do I get there? I think a lot of people consider this as an afterthought at the end of a grant. However, I believe there is much more creativity possible, especially with new media.”* R8 (VUmc). This platform could also offer dissemination support, house tools, and products that serve as a source of inspiration and facilitate knowledge sharing among researchers.

#### In-house connections

Finally, both survey respondents and interviewees suggested the importance of establishing strong in-house connections. This could involve having a dedicated individual or contact within APH who can provide advice and guidance on dissemination. In the survey, a respondent stated: *“Much more help is needed from experts, people with a communication background. They can give advice and help with writing and dissemination. These kinds of activities cost lots of time and are not doable next to a scientific job. But most importantly, we do not have the expertise, we are not trained for these skills. And it is not something you can learn from a two-day course.”* Such a dedicated person could be a general resource available to all researchers, or specific to a research group. This approach could enhance the effectiveness of dissemination efforts and reduce the use of ineffective strategies. Furthermore, participants emphasized the importance of having additional in-house facilities, including information and resources regarding skilled graphic designers, as well as guidance on how to obtain specific materials or tools.

## Discussion

This study explored public health researchers’ approaches, barriers, and needs with regard to dissemination. HBCD researchers reported primarily relying on traditional dissemination approaches, e.g., academic journals (93%, *N* = 39), conferences (93%, *N* = 39), and reports to funders (71%, *N* = 30). Social media (67%, *N* = 28) was also frequently mentioned as a dissemination approach. Dissemination is often prioritized late in projects due to time constraints and competing priorities. Researchers mentioned a lack of time, money, knowledge, and skills but also limited awareness of available support as barriers. A need for more resources, education, and a shift in mindset was expressed, suggesting a comprehensive inspiring platform and stronger in-house connections as solutions.

Among public health researchers in the UK, the use of traditional approaches could be explained by the way in which impact of research is evaluated in the UK, with a strong focus on traditional academic dissemination strategies [11]. Uncontrolled and horizontal methods such as publishing in peer-reviewed journals and presenting at academic conferences [20] could be viewed as forms of communication (‘letting it happen’ [4]) rather than dissemination (‘helping it happen’ [4]). Planned strategies such as through news media, social media, policy briefs, one-on-one meetings, workshops, and seminars [7] are better suited to reach an audience that can create societal change, e.g., practitioners and policymakers [1]. Importantly, the use of traditional dissemination methods is linked to significant costs, including submission fees, article publishing charges (APCs), or open access (OA) charges [21]. Brownson and colleagues [10] plea for a change in how research is funded and how researchers are incentivized, requiring institutions to commit to dissemination for the long term. HBCD researchers in our study emphasized that if active dissemination methods (i.e., tailoring the message and medium to a specific audience using methods such as media engagement, and knowledge brokers [20]) become more integrated into the academic culture, and if they receive adequate time and resources for this, their dissemination efforts could improve and expand beyond the use of traditional approaches.

As a potentially underutilized form of active dissemination, HBCD researchers recognized the potential of social media. Social media can promote interaction between individuals and health organizations by changing the speed and the type of engagement [22]. It can be seen as a cost-effective way to publicly report on a specific health concern, improve communication during public health emergencies and outbreaks, and inform audiences about health issues [22]. Therefore, it is increasingly being used by public health organizations, although public health researchers remain uncertain of how to best use social media for dissemination purposes [22]. Literature shows that using social media as dissemination strategy is significantly associated with more downloads and total number of citations of scientific publications [7]. Knowledge on how and when to effectively use social media for science communication could be implemented in educational initiatives on dissemination practices. Consequently, the use of a comprehensive inspiring platform could offer ideas and serve as a first step in guiding the use of social media when disseminating research findings. Of course, it is important to note that social media dissemination strategies are not suitable for all target audiences, messages, and researchers.

### Strengths and limitations

Strengths of this study include the focus on an underexplored but essential topic and the mixed-methods design which allowed for data triangulation. Within the quantitative method, the use of a preexisting survey enabled the comparison of the results with other literature. For the qualitative data, conducting most interviews in person and including participants from a wide range of career levels and institutions allowed for profound insights and outcomes. Study limitations include the specific context and the low response rate to the survey, limiting the generalizability of the findings. However, the inclusion of qualitative data helped to offset this limitation. Another limitation is the lack of personal details collected such as age, gender, and focus area of research, which would have provided valuable information on whether these personal factors affect dissemination activities. Finally, it is important to acknowledge the presence of bias, including participation bias, selection bias, and the possibility of socially desirable responses given by participants. This may have resulted in an overestimation of the value and importance attributed to dissemination.

### Implications of findings and recommendations for future research

HBCD researchers experienced a lack of institutional support and priority put on dissemination. This lack of institutional support, recognized through researcher function descriptions, assessment forms, and the focus on traditional dissemination strategies was also observed in other studies [12]. Colditz and colleagues [23] propose to change the metrics for promotion and place a more substantial weight on public health impact to improve the dissemination of research. Brownson and colleagues [7] advocate for a shift in academic cultures and incentives that emphasize establishing connections between researchers and research users. Other institutional and structural changes to facilitate change in the public health research setting could include, e.g., restructuring academic performance measures, both within and external to academic institutions, funding agencies creating demand for dissemination, and developing training schemes [23,24]. The systemic changes necessary for a stronger focus on dissemination and implementation in order to improve public health [23] could benefit from a systems analysis on two levels. First of all, the scientific system around dissemination includes forces shaped by interactions among multiple agents, such as funding agencies [25], external parties (i.e., governments, interest groups, commercial organizations), universities, and many more. System thinking tools such as the iterative learning process focused on identifying needs in the system, matching these needs, implementing strategies, evaluating outcomes, and deciding what should be sustained [26], could help determine the best starting points for sustainable system change. It is likely that the academic publishing subsystem, with increasing lack of reviewer capacity and fraudulent publishers exploiting the OA model, plays a significant role [27]. Secondly, the use of a systems perspective can also be considered in the process of dissemination and adaptation of findings. Cuijpers and colleagues [28] cite that the dissemination and adaptation of findings and interventions is often not conducted systematically. Kohatsu and colleagues [29] created the EBPH approach, defined as “*the process of integrating science-based interventions with community preferences to improve the health of populations”* (p.419). This approach recognizes that decisions about public health must take into account important contextual aspects (i.e., political and organizational factors) in addition to research.

Finally, for dissemination to be effective it is ideally a push-pull model, where both researchers and end-users play active roles with a focus on both barriers and facilitators [1]. This study has primarily focused on the barriers of the push side (i.e., adopters with explicit knowledge from research driving an innovation [1]), with limited attention to facilitators and the pull side (i.e., demand among potential end-users [1]).

## Conclusion

HBCD researchers emphasized the importance of dedicated time and budget for dissemination, as well as other forms of institutional support. Overall, there was a need for a shift in mindset, more educational initiatives, greater integration of dissemination into their roles, the establishment of a comprehensive inspiring platform, and stronger in-house connections to support HBCD researchers’ dissemination efforts.

## Supporting information

Nieuwland et al. supplementary material Nieuwland et al. supplementary material

## Supplementary material

The supplementary material for this article can be found at https://doi.org/10.1017/cts.2024.527
