# Supplementary material for: Exploring public health researchers’ approaches, barriers, and needs regarding dissemination: A mixed-methods exploration
Source: J Clin Transl Sci. 2024 Apr 22;8(1):e81. doi: 10.1017/cts.2024.527 (PMC11091924; doi:10.1017/cts.2024.527)
Supplement: Nieuwland et al. supplementary material [file S2059866124005272sup001.docx]

**Supplementary material 1 – Quantitative survey**

**Disseminating the findings of health services and public health research**

This survey aims to find out what steps public health and health services researchers working in the HBCD research program are currently taking to disseminate the findings of their research.

For this project, we are defining dissemination as the broad range of activities used to spread scientific knowledge to a target audience through planned strategies.

The survey contains 26 questions and can be completed in about 15 minutes.

Any information provided will be treated in the strictest confidence and presented on a non-attributed basis.

The survey is part of an APH Voucher project and an internship project for the master: Management, entrepreneurship & policy analysis in the health and life sciences of VU University. The project aims to identify the needs, experiences and approaches of HBCD-researchers when it comes to research dissemination. The knowledge generated from this inquiry can be used within the AmsterdamUMC/APH to set up guidelines and/or policies for dissemination.

The project team comprises of:
 Alicia Nieuwland, Master student VU university;
 Joreintje Mackenbach, Epidemiologist and Assistant professor at AmsterdamUMC;
 Jillian O’Mara, Public health researcher at AmsterdamUMC.

For more details, please contact Alicia Nieuwland ([a.nieuwland@amsterdamumc.nl](mailto:a.nieuwland@amsterdamumc.nl))

**Informed consent**

- Before filling this in please read the information letter attached to the invitation email of the survey.
- I’ve read the information form. I have had to opportunity to ask questions. My questions have sufficiently been answered. I’ve had enough time to decide on my participation in this study.
- I know that participation is voluntary. I also know that I can opt out of the study at any moment without having to give a reason for this.
- I accept to gathering and using the outcomes of the survey for the purpose elaborated on in the information letter.
- I accept to keeping the collected data for up to 5 years within the AmsterdamUMC after this study has finished for control purposes.
- I want to participate in this study.

  Please note that if information becomes known during the study that could affect your consent, the researcher will inform you in good time

**1. Do you accept the term stated above?**

Yes 

No 

**2. I accept to storing my data even after the study has finished for up to 5 years, for possible**

Yes 

No 

**3. Please enter your institution**

AmsterdamUMC location VUmc 

AmsterdamUMC location AMC 

Vrije University Amsterdam (VU) 

University of Amsterdam (UvA) 

**4. Please enter your role/position at this institution**

|  |
| --- |

5**. Is the dissemination of research findings part of your role?**

Yes 

No 

Not sure 

**6. Do you think the dissemination of research findings should be part of your role?**

Yes 

No 

Not sure 

**7. How important to your own research is dissemination?**

Very important 

Important 

Somewhat important 

Not important 

Not sure 

**8. How important is dissemination to the work of your research group?**

Very important 

Important 

Somewhat important 

Not important 

Not sure 

**9. Is there a dedicated person or team responsible for dissemination related activities within your organization and do you make use of the support they offer?**

Yes, there is. And yes I make use of their support 

Yes, there is. I don’t make use of their support 

No, there is not 

Not sure 

**10. Why do you disseminate the findings of your research?**

**Please tick all that apply**

To raise awareness of the findings 

To stimulate discussion/ debate 

To influence policy 

To influence practice 

To transfer research to practice 

To justify public funding 

To attract future funding 

To raise the organizational profile 

To improve your own communication 

To promote public understanding of science 

To satisfy contractual obligations 

Other (please give details below in question 11) 

**11. Please give detail on question 10 is needed.**

|  |
| --- |

**12. Which of the reasons given above for disseminating the findings of your research are the most important?**

Most important ____________________________________

2nd most important ____________________________________

3rd most important ____________________________________

**13. Does your research group have a formal communication/dissemination strategy? Thinks for instance about certain policies and/or guidelines within your research group concerning dissemination.**

Yes 

No 

Not sure 

**14. Do you ever refer to guidance documents or use a framework to plan dissemination-related activities? For example, think about the guidance documents on the APH webpage about dissemination**

Always 

Usually 

Sometimes 

Rarely 

Never 

Not sure 

**15. At what stage in the research process do you usually plan dissemination-related activities?**

When the research is being formulated 

At the proposal stage 

During the research process 

At the draft report stage 

At the final report stage 

At all stages of the process 

Question not applicable 

**16. As part of your research dissemination, do you ever think about who needs to know about the findings and/or who is most likely to be influenced by them or will influence others?**

Always 

Usually 

Sometimes 

Rarely 

Never 

Not sure 

**17. As part of your research dissemination, do you ever consider how audiences or groups you would like to reach access, read, and use research findings?**

Always 

Usually 

Sometimes 

Rarely 

Never 

Not sure 

**18. What methods do you usually use to disseminate research findings?**

**Please tick all that apply**

Academic journals (e.g., BMJ) 

Professional journals (e.g., Pulse) 

Report to funders 

Full report 

Summary report 

Press releases 

Newsletters 

Policy briefing paper 

Email alerts 

RSS feeds 

Targeted mailings 

Conferences 

Seminars and/or workshops 

Face to face meetings 

Networking 

Media interviews 

Social media posts 

Research registers 

Vlogs 

Podcasts 

Posters 

Infographics 

Factsheet 

Other (please give details in question 19) 

**19. Please give details on question 18 if needed.**

|  |
| --- |

**20. Of the methods you use to publish and disseminate the research findings, which do you think generally have the most impact and why?**

|  |
| --- |

**21. Are there any methods of disseminating research findings that you would like to use but are unable to do so?**

Yes 

No 

Not sure 

**22. If you answered yes above, please give details, including what you would need in order to use those methods.**

|  |
| --- |

**23. Is there anything else you can think of that would enhance the impact of your research? If not please type no.**

|  |
| --- |

**24. Do you ever evaluate the impact of your research?**

Always 

Usually 

Sometimes 

Rarely 

Never 

Not sure 

**25. Overall, how do you rate your current research dissemination activities?**

Excellent 

Good 

Adequate 

Poor 

Not sure 

**Thank you for taking the time to complete this survey**

**26. We would like to know how you would want to be informed about the results of this inquiry**

**Please pick all that apply**

Via the HBCD emailing list 

A newsletter or voucher 

On the Amsterdam Public Health Website 

Face-to-face in the form of a presentation 

In a video or vlog format 

In a social media post 

**Supplementary material 2 – Qualitative interview guide**

For the interviews a topic guide containing several exemplary questions per topic are set up. This will help to structure the interview, but also leave space for topics falling outside of the premeditated concepts.

**Opening questions**

- In what way are you familiar with the topic of dissemination of research?

Definition in this study: *to cover the broad range of activities used to spread scientific knowledge to a target audience through planned strategies*’’

- Is research dissemination something that is formally part of role?
- Should research dissemination be formally part of your role?
  - Why do you think should or should not be part of your role?
- How important do you find research dissemination? OR How important is the dissemination of your own research?
- How do you tackle research dissemination? What steps do you follow when disseminating your research
  - How do you experience this process?

**Different parts of the conceptual model**

1. **Problem identification; identify, review, select**

- What leads you to disseminate research findings? What is your motivation behind disseminating research findings?

Think for instance about thing such as: to stimulate a debate, to spread scientific knowledge, to influence policy or for instance more related to justifying public funding or attracting more funding.

- Our these your own reasons (intrinsic) or do they come more from your employer of the organization funding your research?
- How do you determine when and what research needs to be disseminated?
  - Do you determine this, or does somebody else do this? (If so who?)
- What could help to better identify what research needs to be disseminated?

Think for instance about certain tools or guidance that can be offered.

- - Why could these help with the identification?
  - From whom should this guidance come?

1. **Adapt knowledge to local context**

- How do you determine what parts of a research will be disseminated? (How do you determine what fits in the local context?)
- How do you determine what knowledge adopters would want to receive?

Do you for instance talk to them or have other way of findings this out?

- - Why do you determine it this way?
- What influences the adoption of knowledge to the local context?
  - Are there any barriers in this adaptation process?
  - How could these barriers be overcome?
    - Why could this help to overcome barriers?
- How do you experience adopting knowledge to the local context when disseminating your own research findings?

1. **Barriers to knowledge use**

- What barriers do you experience when disseminating your research?
  - Why is this a barrier for you?
- What barriers do you think adopters experience when using research in practice?
  - Why would adopters think this is a barrier?
- How can these barriers be overcome?
- What actions (or guidance) would increase uptake of the knowledge?
  - Why would this increase uptake?
- What could facilitate knowledge use and why? (What do you need as researcher and what would adopters need from you?)

1. **Select tailor and implement dissemination strategies**

- How do you select the right dissemination strategies?
  - What do you need in order to do this?
  - Why is this important?
- What dissemination strategy do you use most often and why?

Think for instance about: publishing in journals, reporting to funders, using newsletters, policy briefings, presenting on conferences, having face to face meetings, using social media or something else.

- - What do you aim to reach with this dissemination strategy, what is the concrete goal of these strategies?
- When do you plan for dissemination of research? (What does this planning normally look like?)

For example, do you do this when the research is being set up, at the end of the research or more during the research.

- - Why do you plan for dissemination at this stage?
- What do you need to effectively plan dissemination strategies?
  - Why would you need this?

1. **Monitor and evaluate knowledge**

- What is the actual impact of dissemination strategies you have used? (What do you reach with it?)
- How do you monitor and evaluate dissemination strategies/plans?
  - Why do you do this? Why is it important to monitor effectiveness?
- How do you measure the effectiveness of research dissemination?
  - Why do you measure it this way?
- What influences the effectiveness of dissemination?
  - Can these factors be steered and how?

1. **Sustain knowledge use**

- Why is it important to sustain knowledge use?
- How can you sustain the knowledge from research dissemination?
- What do you think adopters needs to sustain disseminated knowledge?
- What do HBCD-researchers need to establish this?

**Closing questions**

- How would you describe your current dissemination efforts?

For instance on the scale of: excellent, good, adequate, or poor.

- - Why do you describe it like this?
- Is there anything that we haven’t discussed yet that could help you to enhance the impact of your research?
- Are there any other experiences, need or approaches we haven’t discussed yet, that you want to discuss?

**Thank you for participating in this interview**
